# Cooking for Health: a healthy food budgeting, purchasing, and cooking skills randomized controlled trial to improve diet among American Indians with type 2 diabetes

**DOI:** 10.1186/s12889-021-10308-8

**Published:** 2021-02-15

**Authors:** Caitlin N. Hawley, Corrine M. Huber, Lyle G. Best, Barbara V. Howard, Jason Umans, Shirley A. A. Beresford, Barbara McKnight, Arlette Hager, Marcia O’Leary, Anne N. Thorndike, India J. Ornelas, Meagan C. Brown, Amanda M. Fretts

**Affiliations:** 1 Department of Medicine, University of Washington, 1410 NE Campus Parkway, Seattle, WA 98195 USA; 2 Missouri Breaks Industries Research Inc, 118 S Willow St, Eagle Butte, SD 57625 USA; 3 Medstar Health Research Institute, 6525 Belcrest Rd #700c, Hyattsville, MD 20785 USA; 4 Georgetown and Howard Universities Center for Clinical and Translational Science, 4000 Reservoir Road NW, Washington, DC 20007 USA; 5 Department of Epidemiology, University of Washington, 1410 NE Campus Parkway, Seattle, WA 98195 USA; 6 Department of Biostatistics, University of Washington, 1410 NE Campus Parkway, Seattle, WA 98195 USA; 7 Cheyenne River Sioux Tribe Adult Diabetes Program, 24276 Airport Rd, Eagle Butte, SD 57625 USA; 8 Massachusetts General Hospital and Harvard Medical School, 55 Fruit St, Boston, MA 02114 USA; 9 Department of Health Services, 1410 NE Campus Parkway, Seattle, WA 98195 USA

**Keywords:** Indians, north American, Diabetes mellitus, type 2, Rural population, Randomized controlled trial, Curriculum, Education, distance, Diet, food, and nutrition, Cooking, Budgeting

## Abstract

**Background:**

The prevalence of poor diet quality and type 2 diabetes are exceedingly high in many rural American Indian (AI) communities. Because of limited resources and infrastructure in some communities, implementation of interventions to promote a healthy diet is challenging—which may exacerbate health disparities by region (urban/rural) and ethnicity (AIs/other populations). It is critical to adapt existing evidence-based healthy food budgeting, purchasing, and cooking programs to be relevant to underserved populations with a high burden of diabetes and related complications. The Cooking for Health Study will work in partnership with an AI community in South Dakota to develop a culturally-adapted 12-month distance-learning-based healthy food budgeting, purchasing, and cooking intervention to improve diet among AI adults with type 2 diabetes.

**Methods:**

The study will enroll 165 AIs with physician-diagnosed type 2 diabetes who reside on the reservation. Participants will be randomized to an intervention or control arm. The intervention arm will receive a 12-month distance-learning curriculum adapted from Cooking Matters® that focuses on healthy food budgeting, purchasing, and cooking skills. In-person assessments at baseline, month 6 and month 12 will include completion of the Nutrition Assessment Shared Resources Food Frequency Questionnaire and a survey to assess frequency of healthy and unhealthy food purchases. Primary outcomes of interest are: (1) change in self-reported intake of sugar-sweetened beverages (SSBs); and (2) change in the frequency of healthy and unhealthy food purchases. Secondary outcomes include: (1) change in self-reported food budgeting skills; (2) change in self-reported cooking skills; and (3) a mixed-methods process evaluation to assess intervention reach, fidelity, satisfaction, and dose delivered/received.

**Discussion:**

Targeted and sustainable interventions are needed to promote optimal health in rural AI communities. If effective, this intervention will reduce intake of SSBs and the purchase of unhealthy foods; increase the purchase of healthy foods; and improve healthy food budgeting and cooking skills among AIs with type 2 diabetes – a population at high risk of poor health outcomes. This work will help inform future health promotion efforts in resource-limited settings.

**Trial registration:**

This study was registered on ClinicalTrials.gov on October 9, 2018 with Identifier NCT03699709.

**Supplementary Information:**

The online version contains supplementary material available at 10.1186/s12889-021-10308-8.

## Background

In the United States, there are marked ethnic disparities in the prevalence of type 2 diabetes, and the burden of type 2 diabetes in American Indian (AI) communities is particularly high [1]. AIs are 2.5 times more likely to have a diagnosis of type 2 diabetes than non-Hispanic whites of similar age [2]. Further, AIs with known diabetes have more than double the risk for cardiovascular disease (CVD) than AIs without diabetes [3]. In the Great Plains, where our study is based, type 2 diabetes is the second leading cause of death for AIs, and AIs are 5.5 times more likely to die from type 2 diabetes than Caucasians of similar age [4]. Moreover, AIs with type 2 diabetes in Montana are three times as likely to have CVD than those without diabetes [5].

Results from the Strong Heart Family Study (SHFS), a longitudinal study of risk factors for CVD among 2780 AI adults from 12 rural AI communities in Arizona, Oklahoma, North Dakota, and South Dakota (including the community we are working with for this study), indicate poor diet quality among most participants: 3.8% consumed 4.5+ cups of fruits and vegetables per day; < 1% consumed 2+ servings of fish/week; < 1% consumed 3+ servings of whole grains/day; 13.8% consumed < 1500 mg of sodium/day; 65.3% consumed > 2 servings of processed meat/week; and 71% consumed > 36 oz. of sugar-sweetened beverages (SSBs)/week [6]. As diet quality is a leading risk factor for the development of chronic diseases, including diabetes and CVD, developing interventions in AI communities that focus on achieving current American Diabetes Association (ADA) consensus recommendations for effective diabetes nutrition is warranted [7, 8].

Pilot work that informed the study described herein included conducting four focus groups with community members and seven key informant interviews with stakeholders involved in community nutrition programming to better understand primary barriers and possible facilitators to healthy eating. Results highlighted the need for culturally-adapted healthy food budgeting, purchasing, and cooking skills interventions to help optimize community members’ acquisition and consumption of healthy food on a limited budget [9].

Cooking Matters® is a practice-based cooking and nutrition education curriculum included in the United States Department of Agriculture Supplemental Nutrition Assistance Program - Education (USDA SNAP-Ed) Toolkit [10]. Cooking Matters® comprises 6 weeks of cooking, menu planning, and nutrition education (curriculum is 50% nutrition and 50% cooking). The target audience is adults with school-aged children. All sessions are intended to be interactive and hands-on, and designed to be delivered in-person once per week. Cooking Matters® has shown positive effects on the consumption of healthy food, food-related preferences and behaviors [11], and food budgeting skills [12]. However, implementation of Cooking Matters® is only feasible in communities that have the infrastructure in place to support in-person delivery of the program. Multiple factors limit the utility of Cooking Matters® in rural and AI communities, including lack of teaching kitchens. Further, long travel distances and limited public transportation make attendance at weekly classes challenging for many community members. These barriers to implementing hands-on cooking skills programs in rural AI communities may exacerbate existing health disparities; it is therefore critical to develop and adapt existing healthy food budgeting, purchasing, and cooking programs to meet the needs of these communities.

The purpose of the Cooking for Health Study is to develop a distance-learning-based culturally-adapted healthy food budgeting, purchasing, and cooking intervention, adapted from Cooking Matters®, for AI adults with type 2 diabetes who reside in an AI community in South Dakota, and to test the efficacy of the intervention on: (1) change (from baseline) in self-reported intake of sugar-sweetened beverages (SSBs); and (2) change (from baseline) in the frequency of healthy and unhealthy food purchases. Secondary outcomes include: (1) change (from baseline) in self-reported food budgeting skills; (2) change (from baseline) in self-reported cooking skills; and (3) a mixed-methods process evaluation to assess intervention reach, fidelity, satisfaction, dose delivered, and dose received. As the ADA does not endorse a prescriptive diet for optimal management of diabetes, but rather promotes the consumption a wide variety of nutrient-dense whole foods [7], the intervention curriculum will focus on promoting the consumption of appropriate portion sizes of a wide variety of whole foods, including fruits, non-starchy vegetables, lean meats, and whole grains, and minimizing the consumption of highly processed foods and foods with added sugars-- in line with ADA recommendations. We recognize that there is emerging research to assess the effect of specific diets on diabetes management (e.g. the effect of very-low carbohydrate diets in diabetes management [13-15]), but focusing on specific diets was beyond the scope of this study.

## Methods/design

### Study design

The Cooking for Health Study is a randomized controlled trial (RCT), which will enroll 165 AIs who reside in a reservation community in South Dakota. Participants will be randomized to a 12-month intervention or control arm using a 1:1 randomization scheme. Participants in the intervention arm will complete a 12-month curriculum, which includes 12 distance-learning lessons (i.e., both paper material and videos available through an online learning platform) related to healthy food budgeting, purchasing, and cooking skills. Participants in the control arm will receive access to the intervention materials at the end of the study. The curriculum will be based in social cognitive theory, which posits that to change health behaviors, you must increase self-efficacy to perform the behavior [16]. All study participants will attend three in-person study visits for data collection at baseline (month 0), month 6 and month 12. Laboratory staff who process blood samples and data analysts will be blinded to study arm.

### Study population

American Indian men and women 18–60 years old with a physician-diagnosis of type 2 diabetes [17] who reside on the reservation and self-report doing most of their household’s food shopping and meal preparation will be eligible to participate in the study. Only one person per household will be eligible to participate to avoid non-independence of food choices and potential cross-arm contamination; if more than one eligible household member expresses interest, one will be chosen at random. Individuals who are pregnant, have a history of bariatric surgery, are on dialysis, or are cognitively impaired will be excluded from participation as these conditions may influence diet or ability to engage with the intervention. Additionally, individuals without a reliable place to cook or store food (e.g., homeless) will be ineligible to participate.

### Recruitment strategies

The tribal Adult Diabetes Program, a community-based clinical care program focused on supporting community members with diabetes management, will assist with recruitment. The Adult Diabetes Program will mail letters to eligible patients from their clinic to describe the study and invite participation; the letter will request that interested patients contact study staff directly. Radio announcements, newspaper ads, social media, and flyers posted around the community, as well as solicitation at community events like health fairs and community meetings, will also be used as recruitment strategies.

### Curriculum development

The Cooking for Health Study used Cooking Matters® as a foundation for intervention development. Investigators planned to: (1) modify and supplement the curriculum to use a distance-learning platform (versus standard in-person delivery) to maximize reach in a resource-limited setting; (2) implement a more comprehensive and longer-term curriculum (12 months rather than 6 weeks used by Cooking Matters®); and (3) focus on adults with type 2 diabetes (versus families with school-aged children, the current population on which Cooking Matters® focuses). However, focus groups with community members highlighted the need for more substantial adaptations to the curriculum. Further adjustments included: (1) a greater focus on food budgeting and meal planning for multi-generational families with limited budgets, including how to most effectively use government assistance, such as the Food Distribution Program on Indian Reservations (FDPIR), commonly known as commodity foods, or SNAP; (2) incorporation of healthy, traditional and locally available foods into the curriculum and recipes; (3) more detailed instruction on unit pricing, particularly for individuals with low literacy and numeracy skills; (4) focusing the curriculum on the ADA consensus recommendations for effective diabetes nutrition for management of type 2 diabetes, including limiting unhealthy food and SSBs [7]; (5) food safety, including proper storage of fresh and frozen fruits, vegetables, and meats; and (6) the incorporation of culturally meaningful language, art, and photos throughout the curriculum. All modifications were made following the stages of cultural adaptation, as described by Barrera and Castro [18].

### Final curriculum

The final curriculum that was developed includes 12 lessons (one lesson per month). Each lesson focuses on a specific theme and consists of both paper materials and videos (Table 1), comprising up to one and a half hours of material per month. Videos and paper materials are presented in short segments that take 10–20 min to complete, maximizing curriculum flexibility since they can be reviewed in several short sessions throughout the month. In total, each month’s lesson includes 3–8 short videos (video lengths range from 1 to 16 min) that highlight key points described in the paper materials, as well as recipe demonstrations, budgeting and shopping tips, and visualizations of serving sizes. Most videos were recorded with a community member serving as the instructor. Studies in other communities have shown that intervention effectiveness was maximized when interventionists and participants were ethnically-matched [19]. In focus groups conducted during the intervention development phase of the study, the community expressed the need for ethnic concordance across interventionists and participants.

**Table 1:** Curriculum Topics & Targeted Behavior^a^

Month	Theme	Targeted Behaviors	Videos
1	Introduction	Introduce MyPlate; Essential nutrients; Basic food safety (Clean, Separate, Cook, Chill); Using a food thermometer; Knife skills; Kitchen tools; Cooking terms; Measuring terms/common units; Reading and doubling/adjusting/reducing recipes; Recipes and cooking tips	-Overview of the Month 1 paper materials -Delicious Dips & Spreads -Spice It Up -Pinch & Claw Knife Skills
2	Getting healthy food	Stocking a pantry with staples and commodity foods; Comparing prices and using unit prices; Pros and cons of buying in bulk; Reading Nutrition Facts labels and ingredient lists; Developing and using a shopping list; Recipes and cooking tips	-Overview of the Month 2 paper materials -Chicken & Rice Soup -Corn Bread -How to Measure Dry Ingredients -How to Measure Wet Ingredients -How to Use a Food Thermometer
3	Vegetables	Why eating vegetables is important; Diabetes hints; Nutrients and impacts on health; Vegetables by Color; Daily recommendations for consumption; Common portions; Ways to eat more vegetables; Diabetes tips; Buying and storing fresh vegetables; Weighing and calculating; Seasonality; Food safety; Cooking vegetables in 3 ways; Vegetable substitutions; Recipes and cooking tips; $10.00 Cooking Challenge	-Overview of the Month 3 paper materials -Stir Fry -Sweet Potato Fries -How to Get the Best Deal on Fruits & Vegetables -How to Prepare Winter Squash
4	Fruits	Why eating fruits is important; Diabetes hints; Fruits by color; Nutrients and impacts on health; Ways to eat more fruits; Daily recommendations for consumption; Common portions; Diabetes tips; Buying and storing fresh fruits; Weighing and calculating; Seasonality; Food safety; Fruit substitutions; Recipes and cooking tips	-Overview of the Month 4 paper materials -Apple Salad -Fruit Tart -Choosing Great Bananas -How to Make the Most of Your Bananas
5	Dairy	Why eating dairy foods is important; Diabetes hints; Choosing healthy dairy products; Daily recommendations for consumption; Common portions; Lactose intolerance and substitutions; Tips for maximizing food dollars; Food safety; Cooking dairy; Recipes and cooking tips	-Overview of the Month 5 paper materials -Make a Fruit Smoothie -Choosing Healthy Dairy -Magic Mix -Magic Mix Cream of Anything Gravy -Quesadillas
6	Protein/Meat	Why eating protein is important; Diabetes hints; Lean and low-fat meat; Cholesterol and fat; Nutrients and impacts on health; Daily recommendations for consumption; Common portions; Portion distortion; Ways to eat more protein; How to shop to protein foods; Storage and handling; Food safety; Recipes and cooking tips	-Overview of the Month 6 paper materials -Meatball Master Mix -Sweet & Sour Meatballs -Meatball Sub -Meatball Stroganoff -How to Drain Ground Beef -How to Make a Slow Cooker Meal -Scrambled Egg Muffins
7	Grains	Why eating grains is important; diabetes hints; Nutrients and impacts on health; Refined vs. whole grains; Daily recommendations for consumption; Common portions; How to shop for grains; Choosing whole grains; Food safety; Cooking with grains; Carbohydrate counting; Recipes and cooking tips	-Overview of the Month 7 paper materials -Choosing Whole Grain Bread -Bread In A Bag -Spanish Rice
8	Food budgeting and meal planning	Food wants vs. food needs; Developing a weekly/monthly food budget; Typical costs of food at home in the USA; Meal planning and benefits (time, money, etc.); Planning balanced meals; Tracking income and expenses; Creating a spending plan; How to track food purchases; Following a food budget; Prices at local grocery store to illustrate costs; Recipes and cooking tips; $10.00 Cooking challenge	-Overview of the Month 8 paper materials -Hamburger Casserole -Fast Food vs Fresh Food -How to Plan a Menu -How to Use Planned Overs
9	Empty calories	Empty calories; Sweeteners; Reading nutrition labels (understanding fats and sugars); Alternatives to SSB and foods that contain empty calories; Fats and oils (saturated, unsaturated, and trans fats); Diabetes hints; Lowering intake of empty calories; Lightening up recipes; Recipes and cooking tips	-Overview of the Month 9 paper materials -What’s In Your Drink -What’s In Your Food … Sugar
10	Snacks and eating on the go	Breakfast ideas; Stocking your pantry with healthy snacks; Choosing healthy snacks; Portion size; Tips to eat healthy when time is limited; Tips to eat healthy when traveling; Packaged food makeover; Recipes and cooking tips	-Overview of the Month 10 paper materials -Hummus -Hearty Egg Burritos
11	Traditional foods	Lakota values related to health and well-being; Why eating traditional foods is important; The 4 Food Way using the Medicine Wheel; My Native Plate; Traditional recipes and cooking tips	-Overview of the Month 11 paper materials -Star Boy Video (SD Public TV) -Savor Dakota Timpsila -Savor Dakota Wild and Accessible -Traditional Foods -Savor Dakota: Wojapi
12	Celebrating healthy eating	Hidden sugars and fats; Artificial sweeteners; Low fat substitutions; Celebrating eating healthy; Using healthy flavors (herbs and spices); Healthy cooking for and eating at celebrations; Carbohydrate counting and meal planning with diabetes; Recipes and cooking tips	-Overview of the Month 12 paper materials -10 Doable Ways You Can Enjoy Meals on Special Days -Fry bread recipe
Appendix		Ingredient substitutions; Measuring conversions	

^a^All lessons have been modified with cultural language, art, photos, and localized recipes.

All videos will be available through Canvas®, a highly customizable online distance-learning platform with a simple interface [20]. Importantly, Canvas® records frequency and length of time users log onto the system—which will allow for an objective assessment of intervention reach and dose received. Participants randomized to the intervention arm will be able to watch the videos through Canvas® on their personal computers or mobile devices at home or using internet available in public spaces (e.g. library, tribal community buildings). Tablets will be available for drop-in use at the study field site, the Adult Diabetes clinic, and the tribal field health clinics.

### Informed consent

All research activities were approved by the University of Washington (UW) Institutional Review Board (IRB), the Indian Health Services Great Plains Area IRB, and the tribal health board. Study staff will obtain written informed consent from all study participants before data collection at their first study visit. Study staff will describe all study procedures and the risks and benefits of participation. Study staff will inform potential participants that participation in the study is voluntary, and participants may withdraw at any time. After study staff have addressed any questions or concerns, they will ask the participant to sign the consent form.

### In-person study visits^1^

All study participants will complete in-person study visits at baseline, month 6, and month 12 at the study field site on the reservation. Each in-person study visit includes a personal interview, a physical exam, fasting blood draw, and completion of several questionnaires to ascertain usual (i.e., past 6 months) diet and other diet-related behaviors (e.g. frequency of healthy and unhealthy food purchases, cooking confidence, food resource management, and household food shopping habits). During months 6 and 12, a random subsample of participants in the intervention arm (*n* = 30) will partake in semi-structured interviews.

#### Personal Interview & Physical Exam

During the personal interview, participants will answer questions about their medical history and other current health-related behaviors (e.g., smoking status, alcohol use, physical activity). The study nurse will document type and dosage of current prescription medications. The physical exam will include assessments of body mass index (BMI), waist circumference, and blood pressure. Weight and height will be taken while the participant is standing after removing shoes and heavy objects from pockets. BMI will be calculated as body weight divided by height squared (kg/m^2^). Waist circumference will be measured at the umbilicus while the participant is in a supine position. Blood pressure will be measured three times on the right arm using Omron sphygmomanometers after 5 min rest, and the average of the last two measurements will be recorded.

#### Fasting blood draw

Less than two tablespoons (30 mL) of fasting blood (12 h fast) will be collected and processed on-site with aliquots of serum, plasma, and whole blood stored at − 80 degrees Celsius. All measurements will be made at the Penn Medical Laboratory at MedStar Health Research Institute (MHRI), a College of American Pathologists (CAP) accredited lab [21]. Plasma glucose will be measured using a glucose oxidase method. Insulin will be analyzed using a sensitive immunoassay, and HbA1c will be measured using high-performance liquid chromatography. Total cholesterol will be measured by an enzymatic method. High-density lipoprotein will be measured by cholesterol assay following phosphotungstic acid-magnesium chloride precipitation and cholesterol ester hydrolysis. Low-density lipoprotein cholesterol will be measured by the Friedewald formula, except when triglycerides exceed 400 ml/dl in which case it will be measured directly, all on the Vitros 5.1 platform (Ortho Clinical Diagnostics, Rochester NY) [22].

#### Diet assessment

To estimate usual diet during the past 6 months, participants will complete a Nutrition Assessment Shared Resources (NASR) Food Frequency Questionnaire (FFQ). The NASR FFQ is a widely-used FFQ with demonstrated reliability and validity [23-25]. It has been modified to include foods commonly consumed locally (i.e., fry bread, Indian tacos, and buffalo), in addition to food items on the standard NASR FFQ. For some ethnic groups, the inclusion of a supplement to ascertain the intake of foods commonly consumed in the community on the FFQ produced more accurate nutrient estimates [26]. Usual diet during the past 6 months will be estimated using assessments of consumption frequency (i.e., never/<once per month, 1 per month, 2–3 per month, 1 per week, 2 per week, 3–4 per week, 5–6 per week, 1 per day, and 2+ per day) and portion size (small, medium, or large). Mean daily energy and macronutrient intakes will be calculated for each study participant using the Nutrition Data Systems for Research (NDSR) software v2019 developed by the Nutrition Coordinating Center (University of Minnesota, Minneapolis, MN). The frequency response for each food item on the FFQ and supplementary foods questionnaire will be multiplied by the nutrient content of the documented portion size of the food. The nutrient results will be summed to obtain a measure of total intake [27].

#### Healthy/unhealthy food acquisition

Frequency of healthy and unhealthy food purchases over the past 30 days will be assessed using a modified version of a food acquisition survey that was developed to quantify foods commonly purchased or acquired in another AI community [28]. Participants will report the number of times they purchased 47 foods commonly available in the community during the past 30 days.

#### Cooking confidence

A modified version of the Cooking Confidence Scale [11, 12] will be used to assess confidence in preparing healthy food. For the purposes of this study, the standard Cooking Confidence Scale was modified for clarity based on community input, and includes questions such as “How confident are you that you can wash, cut, and prepare fruits and vegetables?” The instrument employs a Likert-type scale with responses ranging from 1 (not at all confident) to 5 (very confident).

#### Food resource management

A modified version of the Food Resource Management Scale will be used to assess participants’ ability to budget for foods throughout the month [11, 12]. The instrument includes four questions related to shopping behaviors to maximize food resources, such as “How often did you use a grocery list when you went grocery shopping in the past two weeks?” The instrument employs a Likert-type scale with responses ranging from 1 (never) to 5 (always).

#### Semi-structured interviews

**Fig. 1:**
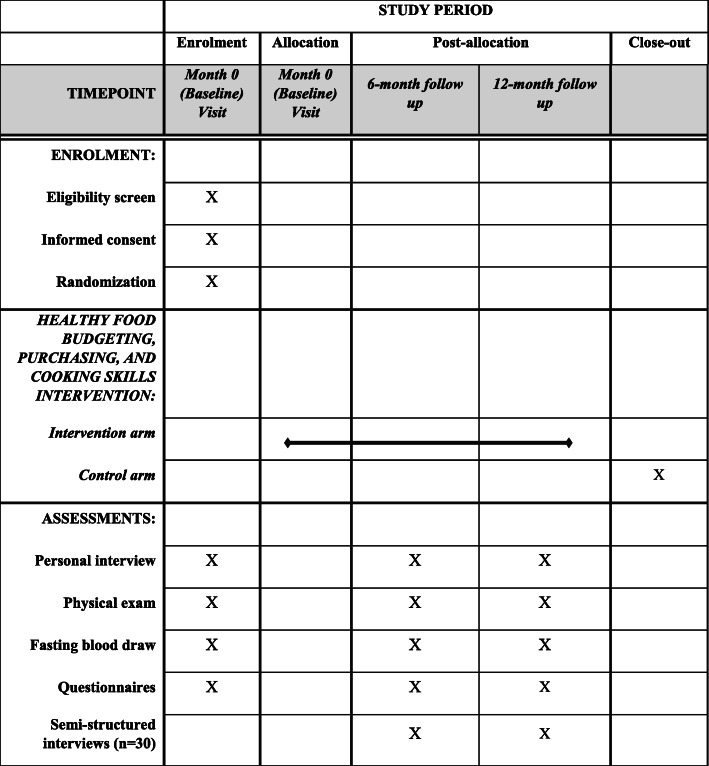
Schedule of enrolment, interventions, and assessments

During months 6 and 12, a random sub-sample of study participants in the intervention arm (*n* = 30) will complete semi-structured interviews by phone to ascertain satisfaction with the intervention, the potential impact of the intervention on their food choices, and feedback on lesson content. Questions were developed using the theoretical framework of acceptability [29], and focus on affective attitude, burden, and perceived effectiveness. An example question is: “Which lessons/videos did you find the most useful?” (Fig. 1)

### Randomization

Investigators will generate a 1:1 randomization sequence using a permuted block design with concealed blocks of variable size. Investigators will provide study staff with sequentially numbered sealed opaque envelopes that contain treatment assignment. After each participant’s baseline data is collected, study staff will open the next-in-order sealed envelope to determine the participant’s arm assignment (i.e., intervention or control).

### Intervention materials

After randomization, participants assigned to the intervention arm will receive all intervention materials, including a binder with all paper materials (i.e., lesson handouts, recipes), a username and password to access videos on Canvas®, and a reusable shopping bag with several tools intended to enhance engagement with the study materials (i.e., calculator, measuring cup set for dry and liquid ingredients, measuring spoon set, stirring/cooking spoons, rubber scraper, turner, whisk, cutting board, knife set, and CalorieKing© Book). Participants randomized to the control arm will receive these materials at the end of the study.

### Incentives

All study participants will receive $100 for each in-person study visit as compensation for time and travel. They will also receive a monthly newsletter by mail highlighting tips for diabetes management and other topics related to diabetes self-care (e.g., stress reduction tips, managing medications, traveling with diabetes). Participants in the intervention arm who watch the videos each month (tracked by study staff through Canvas® and by a log-in book available at the community health clinics) will be entered into a monthly raffle for a cooking-related appliance. The study binders distributed to participants in the intervention arm will contain 12 pre-stamped-and-addressed postcards, one for each lesson, with a question related to that month’s materials. If participants in the intervention arm return the postcard to study staff, they will receive an additional entry in a monthly raffle or a gift card to the local grocery store for healthy food.

### Outcomes

The effect of the intervention on change (from baseline) in self-reported intake of SSBs and frequency of healthy and unhealthy food purchases are the primary outcomes of interest. Secondary outcomes include change (from baseline) in self-reported food budgeting skills and cooking skills, and a process evaluation to enable investigators and staff to evaluate intervention reach, fidelity, participant satisfaction, dose delivered and dose received. This information will guide future iterations of the intervention, as appropriate. Tertiary/exploratory outcomes include change (from baseline) in self-reported intake of fruits and vegetables, whole grains, and legumes; change (from baseline) in food beliefs and attitudes and an alternative assessment of cooking skills; and change in BMI, waist circumference, diabetes control (HbA1c), fasting glucose, diabetes medication usage, high-density lipoproteins, low-density lipoproteins, triglycerides, and blood pressures (systolic blood pressure and diastolic blood pressure) (Table 2).

**Table 2:** Study Outcomes

	Definition	Operationalization	Details
**Primary outcomes**			
Sugar-Sweetened Beverages	Change (from baseline) in self-reported intake (servings/day) of sugar-sweetened beverages	Sugar-sweetened beverages include self-reported intake of fruit drinks, sugar-based energy drinks, and soda. Intake of sugar-sweetened beverages will be estimated using measures of consumption frequency and portion size. Average intakes will be calculated for each study participant using the University of Minnesota Nutrition Data Systems for Research Software by multiplying the frequency response for each beverage on the food frequency questionnaire by the recalled portion size, and then summing for all relevant beverages. Change from baseline with be assessed at 6 months and 12 months (12 months - baseline; 6 months - baseline). As the intervention hopes to decrease intake of sugar-sweetened beverages, lower (i.e., more negative) after - before differences represent a better outcome.	Nutrition Assessment Shared Resource Food Frequency Questionnaire [23]
Healthy and unhealthy food purchases	change (from baseline) in healthy and unhealthy food purchases	Change in healthy and unhealthy food purchases will be estimated using the Healthy/Unhealthy Food Acquisition Survey. The survey includes a list of 47 healthy and unhealthy foods commonly consumed in the community. At each exam (baseline, month 6, month 12), participants will report the number of times he/she acquired each of the 47 foods in the past 30 days. Change from baseline with be assessed at 6 months and 12 months (12 months - baseline; 6 months - baseline). As the intervention hopes to increase the number of healthy food purchases and decrease the number of unhealthy food purchases, higher after - before differences represent a better outcome for healthy foods and lower after - before differences represent a better outcome for unhealthy foods.	The Healthy/ Unhealthy Food Acquisition Survey is a modified version of Dr. Gittelsohn’s Healthy and Unhealthy Food Getting Questionnaire [28]
**Secondary outcomes**			
Food Budgeting Skills	change (from baseline) in food budgeting skills	Change in food budgeting skills will be estimated using the Food Resource Management Scale. The scale includes 4 questions related to shopping behaviors to maximize food resources. The Food Resource Management Scale is a Likert-type scale with responses ranging from 1 (never) to 5 (always). Responses to the four questions will be averaged to create a total Food Resource Management Score. Change from baseline with be assessed at 6 months and 12 months (12 months - baseline; 6 months - baseline). As the intervention hopes to increase food budgeting skills, higher after-before differences represent a better outcome.	The Food Resource Management Scale utilizes 4 questions derived (and adapted for clarity/readability) from Cooking Matters surveys on food resource management [11, 12]
Cooking Skills	change (from baseline) in cooking skills	Change in cooking skills will be estimated using a minor modification to the Cooking Confidence Scale. The Cooking Confidence Scale includes 6 questions related to confidence in preparing healthy foods. It is a Likert-type scale with responses ranging from 1 (not at all confident) to 5 (very confident). Responses to the questions will be averaged. Change from baseline with be assessed at 6 months and 12 months (12 months - baseline; 6 months - baseline). As the intervention hopes to increase cooking skills, higher after - before differences represent a better outcome.	The Cooking Confidence Scale was modified such that one of the six questions from the Cooking Confidence Scale in Cooking Matters [11, 12] was broken up into multiple parts for ease of readability in a low literacy population.
**Process Evaluation Endpoints (Secondary)**			
Intervention Reach		The proportion of those approached that participate in intervention (and the number who subsequently participate) will be used as a marker of intervention reach.	
Intervention Fidelity		The investigators will assess adherence to the study protocol and document barriers and facilitators to implementation throughout the trial.	
Intervention Satisfaction		During the in-person visits at months 6 and 12, a sub-sample of study participants in the intervention arm will meet with study staff for semi-structured interviews to evaluate the overall intervention. Qualitative analyses will assess participant’s satisfaction with the intervention.	
Intervention Dose		*Dose delivered:* number of lessons included in the curriculum available for participants *Dose received:* Number of lessons included in the curriculum completed by participants Dose will be assessed in the intervention arm only	

#### SSBs

Change (from baseline) in self-reported intake (servings/day) of SSBs will be measured using the NASR FFQ [23-25]. SSBs on the FFQ include: fruit drinks fortified with Vitamin C such as Hi-C® or Kool-Aid® (serving size for these SSBs is 1 cup); regular soft drinks (including energy drinks; serving size is 12 oz. or 1 can). Intake of SSBs will be calculated by multiplying the frequency response for each beverage on the FFQ by the portion size, and then summing for all relevant beverages. Change from baseline will be assessed at 6 months and 12 months (12 months – baseline; 6 months – baseline).

#### Healthy and unhealthy food purchases

Change (from baseline) in frequency of healthy and unhealthy food purchases will be assessed using a modified version of the Healthy and Unhealthy Food Acquisition Survey [28]. As the NASR FFQ is unable to adequately discriminate between reported intake of processed foods versus unprocessed foods, healthy and unhealthy food purchases itemized from the modified version of the Healthy and Unhealthy Food Acquisition Questionnaire will be used as a proxy for healthy (i.e., fresh, minimally processed) and unhealthy (i.e., processed) food intake. Foods included on the survey include commonly available and consumed foods in the community. Unhealthy foods on the survey include: white bread; pre-seasoned packaged rice mixes; sugared cereals; bologna, salami, and other sliced/packaged cold lunch meats; frankfurters; hot dogs; canned pork meat product or other regular canned luncheon meats; boxed macaroni & cheese; French fries, hash browns, tater tots, or onion rings; ramen noodles or other cup noodles; chips; prepackaged frozen breaded chicken, chicken strips and chicken nuggets; pizza rolls or frozen pizza; microwavable/prepared meals; regular soda; and lemonade, sports drinks, and energy drinks. Healthy foods on the survey include: whole wheat, multi-grain, or other whole grain bread; rice (white or brown – whole kernel); high fiber cereals (like oatmeal or any bran cereal); peanut butter; milk (1% or skim); bananas; apples and pears (fresh, frozen, or canned); oranges, tangerines, lemons, and grapefruit (fresh, frozen, or canned); berries, cherries, and grapes (fresh, frozen, or canned); any other fresh fruits such as kiwi, plums, apricots, and peaches (fresh, frozen, or canned); dried fruit (including raisins and prunes); mixed vegetables (fresh, frozen or canned); carrots, including baby carrots (fresh, frozen, or canned); corn, including on the cob and kernels (fresh, frozen, or canned); celery; tomatoes, spaghetti sauce, and tomato sauce (fresh, frozen, or canned); lettuce (including salad mix and kits); pumpkin, squash, and zucchini (fresh, frozen, or canned); potatoes (fresh); green beans (fresh, frozen, or canned); peas (fresh, frozen, or canned); cucumber; chicken/turkey – no breading (fresh, frozen, or canned); beans, such as baked beans, pinto beans, and black beans (dried or canned); deer/venison (fresh, frozen, or canned); buffalo (fresh, frozen, or canned). The total number of healthy and unhealthy food purchased over the past 30 days will be defined as the sum of the number of healthy and unhealthy food purchased during that timeframe. Change from baseline will be assessed at 6 months and 12 months (12 months – baseline; 6 months – baseline).

#### Food budgeting skills

Change (from baseline) in self-reported food budgeting skills will be assessed using responses to four questions that comprise a modified version of the Food Resource Management Scale [11, 12]. Responses will be assessed individually as well as averaged to create a total Food Resource Management Score. Change from baseline will be assessed at 6 months and 12 months (12 months – baseline; 6 months – baseline).

#### Cooking skills

Change (from baseline) in self-reported cooking skills will be estimated using responses to eight questions that comprise a modified version of the Cooking Confidence Scale [11, 12]. Responses will be assessed individually as well as averaged to create a Cooking Confidence Score. Change from baseline will be assessed at 6 months and 12 months (12 months – baseline; 6 months – baseline).

#### Intervention process evaluation

As defined below, intervention reach, fidelity, satisfaction, dose delivered, and dose received will be evaluated as process endpoints. Reach will be defined as the proportion of community members approached to participate in the study who enroll and subsequently participate in the study. Fidelity will be assessed by study staff through the documentation of adherence to study protocol and barriers/facilitators to implementation of the study. Intervention satisfaction will be evaluated using semi-structured interviews among a random sub-sample of participants in the intervention arm (*n* = 30) at month 6 and month 12. Intervention dose delivered will be defined as the total number of lessons available to participants in the intervention arm throughout the study period, and intervention dose received will be defined as the number of lessons watched by participants randomized to the intervention arm.

### Statistical approach

#### Power

Power analyses assessed the ability to detect an effect on primary outcomes between baseline and month 12. One hundred sixty-five individuals were estimated to be eligible and willing to participate in the trial. Loss to follow-up during the study is estimated to be 10%. Conservative power estimates are therefore based on a smaller sample size of 150 (75 per study arm) and a Bonferroni correction for two primary outcomes. To minimize repetition, power will be illustrated using one primary outcome of interest: change (from baseline) in self-reported intake of SSBs. The study is estimated to have more than 80% power to detect differences across the one-year trial between the two arms of between 0.029 and 0.34 servings per day depending on the correlation between baseline and month 12 measures (correlations range from 0 to 0.75). This is based on an estimated standard deviation (SD) of 0.4 servings of SSBs per day—an estimate derived from another on-going study on healthy diet and avoidance of SSBs.

##### Statistical analyses

Baseline characteristics of study participants will be examined using descriptive statistics to assess potential differences between study arms, using any baseline characteristics that differ as covariates in secondary analyses. As participants who complete the study may be different than those who drop-out of the study, analyses will compare baseline characteristics between these groups. Data will be reviewed regularly to look for data entry errors and extreme outliers.

Intent-to-treat analyses will estimate the effect of the intervention on all outcomes of interest, compared to control, longitudinally across groups using general linear models. Primary analyses for all outcomes will compare 12-month minus baseline and 6 months minus baseline differences between the intervention and control arms at the end of the study. Missing data will be handled using multiple imputations and/or weighted estimation methods using all available data for imputation to reduce biases, as recommended by the National Research Council Committee in National Statistics Panel on Handling Missing Data in Clinical Trials [30]. A Bonferroni correction will be used to adjust for multiple comparisons (based on 5 primary and secondary non-process evaluation outcomes: *p* = 0.05/5 = 0.01). Exploratory sub-group analyses will be performed, stratified by sex and BMI, to better understand if these factors influence the effectiveness of the intervention.

Descriptive statistics will report quantitative process outcomes. All qualitative assessments related to intervention fidelity and satisfaction, including the semi-structured interviews, will be transcribed, and uploaded into Atlas.ti 8. The principal investigator will work with the research team to develop the coding scheme using health behavior theory and existing literature. Two research assistants will code the transcripts. Investigators will meet with the two coders regularly, and both research assistants will independently code a randomly selected set of transcripts to ensure consistency [31]. The research team will assess recurring themes as part of the process evaluation.

### Dissemination

At the end of the study, investigators will meet with the Tribal Health Committee to report results and discuss possible ways to continue the study (e.g., apply for a grant to include other indicators of CVD; implement the intervention within the Adult Diabetes Program or another tribal program; modify intervention to expand target population). The results of the work will also be presented to the community using REDTalks—a TED-talk-like platform for disseminating ideas and research to AI communities. If the intervention is effective, we will create a “toolbox” that includes all study materials that can be downloaded for use in other tribal communities interested in the curriculum.

### Data safety, confidentiality, and monitoring

#### Data management

REDCap®, a secure web application for building and managing online surveys and databases, will be used for data capture of all questionnaires using password-protected computers [32]. All data will be transferred, encrypted, and backed-up daily, except the NASR FFQ—which will be sent to the Fred Hutchinson Cancer Research Center (Seattle, WA) for analysis. Study staff will transfer other study materials, including transcripts from interviews, to UW by scanning and uploading the documents to a private server using password-protected computers. UW staff blinded to study arm will review all REDCap® surveys and uploaded materials for completeness.

#### Data safety monitoring

Investigators will provide study oversight, ensure that the trial is conducted according to the study protocol, and will be responsible for the data and safety monitoring by ensuring the safety of all participants. As the risks of participating in the study are minimal, we do not anticipate numerous adverse events (AEs). All AEs will be reported according to the UW IRB and the Great Plains Area Indian Health Services IRB.

## Discussion

While numerous obesity, type 2 diabetes, and CVD prevention interventions for adults have been implemented in clinical settings in AI communities [28, 33–38], most focus on diet education or structured physical activity [33–35, 37, 38] in AIs without type 2 diabetes. Though valuable, these curricula do not address the unique challenges of consuming a diet in line with ADA recommendations [39–41] or underlying contextual factors that inhibit individuals’ ability to consume whole foods—including limited budgeting and cooking skills and low literacy and numeracy when purchasing foods. The Cooking for Health Study was developed to help address this pressing need.

Studies in other populations have demonstrated that budgeting and cooking skills influence diet outcomes. Several observational and quasi-experimental studies [11, 42–49] and one RCT [50] have shown positive relationships between cooking skills or in-person cooking training and diet quality in adults [51-58]. Quasi-experimental studies that examined the effect of Cooking Matters® on diet quality indicate that individuals who participated in Cooking Matters® reported consuming more vegetables, low sodium foods, and low-fat dairy products 6 months after completion when compared to participants who did not participate in the program [11]. Additionally, participants who completed Cooking Matters® programming were 17% more confident in managing a monthly food budget when compared to individuals who did not participate in the program [12]. Unfortunately, all studies of the effectiveness of Cooking Matters® used a pre-post design, and the program has not been tested in a randomized-controlled setting. Quasi-experimental studies have well-known limitations, including lack of random assignment to the intervention, difficulty controlling for potential confounding factors that may influence participation in the intervention and diet and health outcomes, and the inability to draw causal inference from results [59]. The Cooking for Health Study will utilize a rigorous experimental design (RCT) to test the effect of a healthy food budgeting, purchasing, and cooking skills intervention adapted from Cooking Matters® on diet and other food-related behaviors.

Community-based participatory research (CBPR) is an approach that uses community engagement and social action to increase health equity in translational research [60]. The core principles of CBPR call for recognizing a community’s identity and culture, building on strengths and resources within the community, and employing collaborative partnerships at all phases of the research [61]. Programs using CBPR have shown sustainability, equity [62–66] and effectiveness [67, 68]. The Cooking for Health Study utilizes CBPR principles at every stage of development/implementation, including in the design of the study to address community desires and needs; leveraging existing community programs/resources; and fostering academic-community relationships to maximize sustainability and reach. All aspects of the Cooking for Health Study were developed based on community input from stakeholders and then modified extensively according to feedback received from community stakeholders and focus groups with community members (more detail on how this process informed the final target outcomes in Additional File 1).

This study has several strengths. First, the RCT-design and use of instruments that have demonstrated reliability and validity will ensure that results generated by this study are robust and that bias is minimized. Second, this study is the result of an academic-community partnership. Developing new research efforts that use existing resources may increase efficiency and the long-term sustainability of the intervention and targeted behavior change. Additionally, working with the local community will make shared responsibility and ownership of the study possible and facilitate an on-going partnership. Finally, the implementation of a healthy food budgeting, purchasing, and cooking skills intervention using distance-learning technology may minimize participant burden and maximize study reach.

This study is not without limitations. Given the study timeline, it is not feasible to assess the long-term sustainability (i.e., post-1 year) of the intervention and potential subsequent behavior change**.** Additionally some participants might not accurately report type, frequency and/or portion size of foods consumed or acquired on the FFQ and food acquisition survey due to recall bias or social-desirability bias—which may influence our ability to detect an effect of the intervention. Finally, diet quality is not the only determinant of optimal diabetes management, and other factors, such as physical activity and medication adherence, also influence diabetes control. Targeting these factors is beyond the scope of the study, although these factors will be assessed.

There is a critical need to develop novel, targeted, and sustainable interventions to promote healthy diet in rural AI communities. In partnership with the community, the work described herein will develop and test the effect of a culturally-adapted intervention to improve diet among AIs with type 2 diabetes. If successful, the intervention can be tailored to other rural and underserved communities. The results of this study will also inform further efforts to design and implement diet interventions in AI communities or other resource-limited settings.

## Supplementary Information

**Additional file 1:** is available in .pdf format. The table lists originally proposed study outcomes (developed by academic investigators) and the final study outcomes that were included in the study based on community input with justification for changes.

## Data Availability

Not applicable.

## Abbreviations

AE: Adverse event
ADA: American Diabetes Association
AI: American Indian
BMI: Body mass index
CVD: Cardiovascular disease
CAP: College of American Pathologists
CBPR: Community-based participatory research
FDPIR: Food Distribution Program on Indian Reservations
FFQ: Food frequency questionnaire
HbA1c: Hemoglobin A1C
IRB: Institutional Review Board
MHRI: MedStar Health Research Institute
NASR: Nutrition Assessment Shared Resources
NDSR: Nutrition Data Systems for Research
RCT: Randomized controlled trial
SD: Standard deviation
SHS: Strong Heart Study
SHFS: Strong Heart Family Study
SSB: Sugar-sweetened beverage
USDA SNAP-Ed: United States Department of Agriculture Supplemental Nutrition Assistance Program – Education
UW: University of Washington

## Footnotes

1 These procedures may need to be adapted due to restrictions related to COVID-19.
