# Supplementary material for: Cooking for Health: a healthy food budgeting, purchasing, and cooking skills randomized controlled trial to improve diet among American Indians with type 2 diabetes
Source: BMC Public Health. 2021 Feb 15;21:356. doi: 10.1186/s12889-021-10308-8 (PMC7883757; doi:10.1186/s12889-021-10308-8)
Supplement: Supplementary file 1 — Additional file 1: is available in .pdf format. The table lists originally proposed study outcomes (developed by academic investigators) and the final study outcomes that were included in the study based on community input with justification for changes. [file 12889_2021_10308_MOESM1_ESM.docx]

| **Additional File 1: Change in Originally Proposed Outcomes with Justifications^1^** | | |
| --- | --- | --- |
| **Originally Proposed Study Outcomes** | **Final Study Outcomes (Based on Community Input--Focus Groups, and Meeting with Community Stakeholders)** | **Rationale for Change** |
| Primary Outcome: change (from baseline) in self-reported intake (servings/day) of sugar-sweetened beverages (measured using the Nutrition Assessment Shared Resource Food Frequency Questionnaire) at 6 months and 12 months | 1. Primary Outcome: change (from baseline) in self-reported intake (servings/day) of sugar-sweetened beverages (measured the Nutrition Assessment Shared Resource Food Frequency Questionnaire) at 6 months and 12 months |  |
| Primary Outcome: change (from baseline) in self-reported intake (grams/day) of processed foods (measured using the Nutrition Assessment Shared Resource Food Frequency Questionnaire) at 6 months and 12 months | 1. Primary Outcome: change (from baseline) in healthy and unhealthy food purchases (measured using a modified version of the Healthy/Unhealthy Food Acquisition Survey) at 6 months and 12 months | As the Nutrition Assessment Shared Resource Food Frequency Questionnaire is unable to adequately discriminate between reported intake of processed foods versus unprocessed foods, healthy and unhealthy food purchases itemized from the modified version of the Healthy and Unhealthy Food Acquisition Questionnaire will be used as a proxy for healthy (i.e., fresh, minimally processed) and unhealthy (i.e., processed) food intake. |
| Primary Outcome: change (from baseline) in food budgeting skills (measured using the Food Resource Management Scale) at 6 months and 12 months | 1. Secondary Outcome: change (from baseline) in food budgeting skills (measured using a modified version of the Food Resource Management Scale) at 6 months and 12 months | This has been changed to a secondary outcome to make the intervention simpler and more focused as the community was primarily interested in diet change.  The original questionnaire was modified based on community input to improve clarity. |
| Primary Outcome: change (from baseline) in cooking skills (measured using the Cooking Confidence Scale) at 6 months and 12 months | 1. Secondary Outcome: change (from baseline) in cooking skills (measured using a modified version of the Cooking Confidence Scale) at 6 months and 12 months | This has been changed to a secondary outcome to make the intervention more focused and clearer for the community, which was primarily interested in diet change.  The original questionnaire was modified based on community input to improve clarity. |
| Primary Outcome: change (from baseline) in healthy and unhealthy food purchases (measured using the Healthy/Unhealthy Food Acquisition Survey) at 6 months and 12 months |  | This outcome was incorporated into primary outcome #2 above. |
| Secondary Outcome: change (from baseline) in self-reported intake (servings/day) of fruits and vegetables (measured using the Nutrition Assessment Shared Resource Food Frequency Questionnaire) at 6 months and 12 months | 1. Tertiary/Exploratory Outcome: change (from baseline) in self-reported intake (servings/day) of fruits and vegetables (measured using the Nutrition Assessment Shared Resource Food Frequency Questionnaire) at 6 months and 12 months | This has been changed to a tertiary/exploratory outcome to make the intervention more focused and clearer for the community, which was primarily interested in lowering intake of sugary drinks and processed foods. |
| Secondary Outcome: change (from baseline) in self-reported intake (grams/day) of whole grains (measured using the Nutrition Assessment Shared Resource Food Frequency Questionnaire) at 6 months and 12 months | 1. Tertiary/Exploratory Outcome: change (from baseline) in self-reported intake (grams/day) of whole grains (measured using the Nutrition Assessment Shared Resource Food Frequency Questionnaire) at 6 months and 12 months | This has been changed to a tertiary/exploratory outcome to make the intervention more focused and clearer for the community, which was primarily interested in lowering intake of sugary drinks and processed foods. |
| Secondary Outcome: change (from baseline) in self-reported intake (grams/day) of legumes (measured using the Nutrition Assessment Shared Resource Food Frequency Questionnaire) at 6 months and 12 months | 1. Tertiary/Exploratory Outcome: change (from baseline) in self-reported intake (grams/day) of legumes (measured using the Nutrition Assessment Shared Resource Food Frequency Questionnaire) at 6 months and 12 months | This has been changed to a tertiary/exploratory outcome to make the intervention more focused and clearer for the community, which was primarily interested in lowering intake of sugary drinks and processed foods. |
| Secondary Outcome: change (from baseline) in cooking skills (using alternate scale—Healthy Food Preparation Scale) at 6 months and 12 months | 1. Tertiary/Exploratory Outcome: change (from baseline) in cooking skills (using alternate scale— a modified version of the Healthy Food Preparation Scale) at 6 months and 12 months | Cooking Skills will primarily be assessed using secondary outcome #4 above. We will explore an alternative method to assess cooking skills using a modified version of the Healthy Food Preparation Scale as a tertiary/exploratory outcome. This has been changed to a tertiary/ exploratory outcome to make the intervention more focused and clearer for the community, which was primarily interested in diet change.  The original questionnaire was modified based on community input to improve clarity. |
| Secondary Outcome: change (from baseline) in food beliefs and attitudes at 6 months and 12 months | 1. Tertiary/Exploratory Outcome: change (from baseline) in food beliefs and attitudes at 6 months and 12 months | This has been changed to a tertiary/exploratory outcome to make the intervention more focused and clearer for the community, which was primarily interested in diet change.  The original questionnaire was modified based on community input to improve clarity. |
| Secondary Outcome: change (from baseline) in body mass index (kg/m^2^) at 6 months and 12 months | 1. Tertiary/Exploratory Outcome: change (from baseline) in body mass index (kg/m^2^) at 6 months and 12 months | This has been changed to a tertiary/exploratory outcome to make the intervention more focused and clearer for the community, which was primarily interested in diet change. |
| Secondary Outcome: change (from baseline) in waist circumference (cm) at 6 months and 12 months | 1. Tertiary/Exploratory Outcome: change (from baseline) in waist circumference (cm) at 6 months and 12 months | This has been changed to a tertiary/exploratory outcome to make the intervention more focused and clearer for the community, which was primarily interested in diet change. |
| Secondary Outcome: change (from baseline) in diabetes control (measured with hemoglobin A1c (%)) at 6 months and 12 months | 1. Tertiary/Exploratory Outcome: change (from baseline) in diabetes control (measured with hemoglobin A1c (%)) at 6 months and 12 months | This has been changed to a tertiary/exploratory outcome to make the intervention more focused and clearer for the community, which was primarily interested in diet change. |
| Secondary Outcome: change (from baseline) in fasting glucose (mg/dl) at 6 months and 12 months | 1. Tertiary/Exploratory Outcome: change (from baseline) in fasting glucose (mg/dl) at 6 months and 12 months | This has been changed to a tertiary/exploratory outcome to make the intervention more focused and clearer for the community, which was primarily interested in diet change. |
| Secondary Outcome: change (from baseline) in diabetes medication usage at 6 months and 12 months | 1. Tertiary/Exploratory Outcome: change (from baseline) in diabetes medication usage at 6 months and 12 months | This has been changed to a tertiary/exploratory outcome to make the intervention more focused and clearer for the community, which was primarily interested in diet change. |
| Secondary Outcome: change (from baseline) in high density lipoproteins (mg/dL) at 6 months and 12 months | 1. Tertiary/Exploratory Outcome: change (from baseline) in high density lipoproteins (mg/dL) at 6 months and 12 months | This has been changed to a tertiary/exploratory outcome to make the intervention more focused and clearer for the community, which was primarily interested in diet change. |
| Secondary Outcome: change (from baseline) in low density lipoproteins (mg/dL) at 6 months and 12 months | 1. Tertiary/Exploratory Outcome: change (from baseline) in low density lipoproteins (mg/dL) at 6 months and 12 months | This has been changed to a tertiary/exploratory outcome to make the intervention more focused and clearer for the community, which was primarily interested in diet change. |
| Secondary Outcome: change (from baseline) in triglycerides (mg/dL) at 6 months and 12 months | 1. Tertiary/Exploratory Outcome: change (from baseline) in triglycerides (mg/dL) at 6 months and 12 months | This has been changed to a tertiary/exploratory outcome to make the intervention more focused and clearer for the community, which was primarily interested in diet change. |
| Secondary Outcome: change (from baseline) in blood pressures (i.e., systolic blood pressure and diastolic blood pressure) at 6 months and 12 months | 1. Tertiary/Exploratory Outcome: change (from baseline) in blood pressures (i.e., systolic blood pressure and diastolic blood pressure) at 6 months and 12 months | This has been changed to a tertiary/exploratory outcome to make the intervention more focused and clearer for the community, which was primarily interested in diet change. |
| **Process Endpoints** | | |
| **Originally Proposed Process Endpoints** | **Final Process Endpoints** | **Rationale for change** |
| Secondary Outcome: intervention reach | 1. Secondary Outcome: intervention reach | No change |
| Secondary Outcome: intervention fidelity | 1. Secondary Outcome: intervention fidelity | No change |
| Secondary Outcome: intervention satisfaction (among those in the intervention arm) | 1. Secondary Outcome: intervention satisfaction (among those in the intervention arm) | No change |
| Secondary Outcome: intervention dose delivered (i.e., number of lessons included in the curriculum available for participants) | 1. Secondary Outcome: intervention dose delivered (i.e., number of lessons included in the curriculum available for participants) | No change |
| Secondary Outcome: intervention dose received (i.e., number of lessons included in the curriculum completed by participants) | 1. Secondary Outcome: intervention dose received (i.e., number of lessons included in the curriculum completed by participants) | No change |

^1^ The focus of the study and study outcomes were modified during the community engagement phase of the study based on community input. Final outcomes more clearly reflect the interests and needs of the community than the study outcomes originally proposed (before community engagement).
